# Oral lichen planus versus epithelial dysplasia: difficulties in diagnosis

**DOI:** 10.1016/S1808-8694(15)30523-1

**Published:** 2015-10-18

**Authors:** Fernando Augusto Cervantes Garcia de Sousa, Thaís Cachuté Paradella, Adriana Aigotti Haberbeck Brandão, Luiz Eduardo Blumer Rosa

**Affiliations:** 1 MSc on Oral Biopathology, Surgeon Dentist; 2 PhD on Oral Biopathology, Surgeon Dentist; 3 Professor, PhD on General Pathology, FOSJC/UNESP, MD; 4 Adjunct Professor of Oral Pathology, FOSJC/UNESP, Surgeon Dentist

**Keywords:** epithelium, lichen planus, mouth mucosa

## Abstract

Histopathological diagnosis of oral lichen planus is not easy since some cases of epithelial dysplasia may present traits which are very similar to those from lichen planus.

**Aim:**

to compare cell alterations which suggest malignancy present in oral lichen planus with those from epithelial dysplasia.

**Material and methods:**

histological cross-sections of oral lichen planus and dysplasia, dyed by hematoxylin-eosin, were analyzed by means of light microscopy.

**Results:**

variance analysis (alpha=5%) revealed a statistically significant difference between the average number of cell alterations in the lichen planus (5.83±1.61) and epithelial dysplasia (4.46±1.26). The chi-squared test did not show statistically significant differences between oral lichen planus and epithelial dysplasia in relation to the following cell alterations: increase in nucleus/cytoplasm ratio, nuclear hyperchromatism, irregular chromatin distribution and enlarged nuclei (p>0.05).

**Conclusion:**

Some cell alterations which suggest malignancy present in the oral lichen planus may also be found in epithelial dysplasia, impairing its diagnosis and, consequently, stressing the importance of following these patients in the long run.

## INTRODUCTION

Lichen planus is one of the most common dermatologic diseases to involve the oral cavity, with prevalence rates ranging from 0.5% to 2% in the general population. Although it is relatively common, oral lichen planus is fraught with controversy, mainly in relation to the possibility of it becoming a malignant condition.^1^, ^2^, ^3^, ^4^

Studies carried out in a number of countries found a considerably high likelihood of injuries initially diagnosed as lichen planus becoming malignant lesions along the years.^4^, ^5^, ^6^, ^7^, ^8^, ^9^ However, some authors believe that most cases of evolution to malignant injury described in the literature are due to incorrect initial diagnosis.^4^,^10^, ^11^, ^12^, ^13^, ^14^, ^15^

Indeed, some cases of epithelial dysplasia may have histopathology findings similar to lichen planus, in a condition known as lichenoid dysplasia. This disease could be easily mistaken for lichen planus and presents true potential to evolve to malignancy.^12^,^13^,^15^,^16^ According to Lodi et al.^15^ (2005), the very chronic inflammatory process present in oral lichen planus may lead to the appearance of cell disorders similar to the alterations seen in epithelial dysplasia, turning diagnosis into an even harder task.

Van der Meij and Van der Waal^17^ (2003) wrote well about such difficulty. The authors observed that in 42% of the cases in which there was agreement upon disease clinical diagnosis, there was no consensus in regards to histopathology diagnosis. On the other hand, in 50% of the cases in which there was such consensus, clinical agreement was never reached.

Additionally, other conditions may present clinical and histopathology characteristics similar to oral lichen planus such as lichenoid reactions, lupus erythematosus, leukoplakia, erythroleukoplakia, and proliferative verrucous leukoplakia.^15^

However, it is the cases of lichenoid dysplasia that mostly concern general practitioners and pathologists, as they present the same traits found in lichen planus combined with varying degrees of epithelial dysplasia. Some authors believe that lichenoid dysplasia should be considered as an injury with high likelihood of evolving to malignancy^13^,^15^,^18^, reinforcing even further the theory that most cases of lichen planus with possible evolution to malignancy stem from failed initial diagnosis.^4^,^10^, ^11^, ^12^, ^13^, ^14^, ^15^

In such context, this study aims to compare cell disorders indicative of malignancy present in oral lichen planus and epithelial dysplasia and inform general practitioners and pathologists of the difficulties surrounding the histopathology diagnosis of lichen planus and the importance of establishing long term follow-up for this group of patients.

## MATERIALS AND METHOD

Our study included 28 cases of oral lichen planus and another 28 patients with epithelial dysplasia (eight mild, 16 moderate and 4 severe cases) diagnosed at a reference service in oral diseases located in São José dos Campos, SP, Brazil, between 1995 and 2005, whose clinical findings and evolution left no doubt as to the diagnosis.

The oral lichen planus cases were reassessed by three independent examiners, following the histopathology criteria set by Eisenberg1^3^ (2000) (Table 1) to confirm initial diagnosis. The cases of epithelial dysplasia were reviewed under the criteria defined by Bánóczy & Csiba^19^ (1976) (Table 2). Doubtful cases were immediately discarded and replaced. Patients exposed to or carrying risk factors for oral cancer such as smoking and drinking were also excluded.

**Table 1:** Histological diagnostic criteria for oral lichen planus.^13^

Key findings
• Liquefaction of the basal layer • Intense lymphocytic infiltrate underlying the epithelium with deletion of the basal layer • Normal epithelial cell maturation pattern **Other findings (not essential)** • Interpapillary crests with serrated edges • Hyperparakeratosis • Civatte bodies • Epithelium separated from lamina propria

**Table 2:** Histological diagnostic criteria for epithelial dysplasia.^19^

Epithelial atypias
• Cell and nucleus pleomorphism • Loss of epithelial stratification • Nuclear hyperchromatism • Multinucleate cells • Increased nuclear-cytoplasmic ratio • Enlarged nucleoli • Thickened nuclear membrane • Duplication of basal layer • Keratinization of individual cells or cell groups in the prickle-cell layer or in deeper layers • Drop-shape projection of epithelial cones • Mitotic figures in the median portion of the epithelium • Atypical mitosis • Increase on mitotic figures • Loss of cell cohesion • Loss of polarity in basal cells718

Tissue specimens stained with H&E were observed in a microscope by two independent examiners. Examiners looked at cell disorders indicative of malignancy and took the following criteria into account: a) increased nuclear-cytoplasmic ratio; b) nuclear hyperchromatism; c) irregular chromatin distribution; d) thickened nuclear membrane; e) loss of cell cohesion; f) enlarged nucleoli; g) multinucleate cells; h) cell/nucleus pleomorphism. Cases in which there was disagreement between examiners were taken to a third tie-breaking examiner.

Gathered data sets were statistically treated using analysis of variance (ANOVA) followed by Tukey's test when required, and the chi-square test, all at a significance level of 5%.

This study was approved by the local research ethics committee under permit 008/2006-PH/CEP as of March 14, 2006.

## RESULTS

All cases of oral lichen planus had basal layer liquefaction and intense lymphocytic infiltrate underlying the epithelium. Inversely, in none of the cases any changes were found in the normal maturation pattern of epithelial cells. In 96.43% of the cases varying degrees of hyperkeratosis were found. Although saw interpapillary crests with serrated edges were not seen, they were altered in 89.29% of the cases. Separation of epithelium from lamina propria and Civatte bodies were observed in only 3.57% of the cases.

Regardless of the degree of epithelial dysplasia, the most frequently observed atypias were duplication of the basal layer (100.00%), loss of basal cell polarity (100.00%) and nuclear hyperchromatism (46.43%). In contrast, keratinization of individual cells or cell groups in the prickle-cell layer or in deeper layers, mitotic figures in the median portion of the epithelium, and atypical mitosis were not seen at all.

In terms of cell disorders indicative of malignant disease, oral lichen planus had an average 5.83±1.61 disorders per case, while epithelial dysplasia cases averaged 4.46±1.26. However, analysis of variance (ANOVA) showed no statistically significant differences between the mean number of cell disorders indicative of malignant disease found in both lesions (p<0.05).

**Figure 1:**
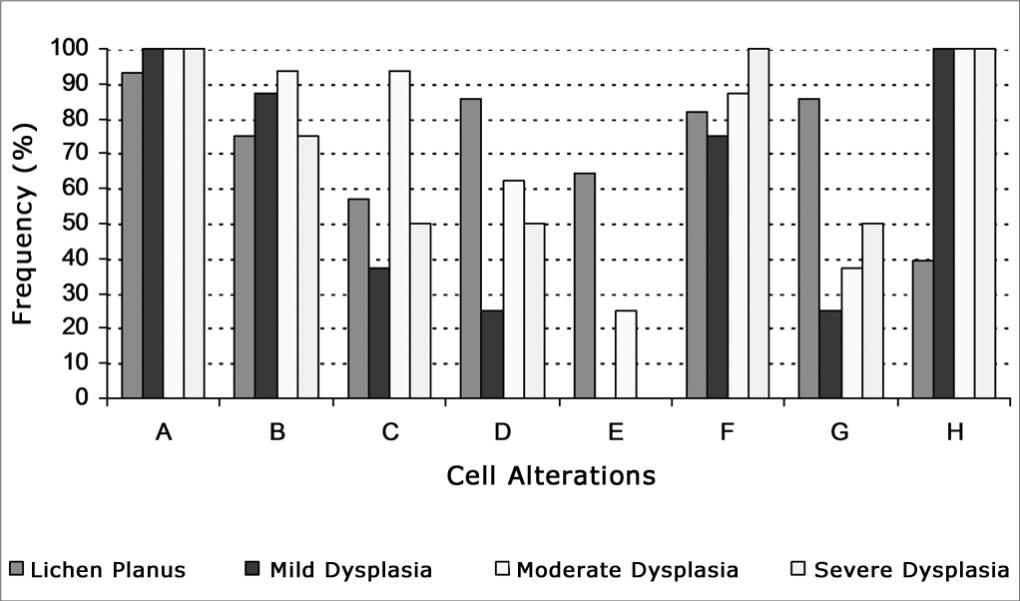
Frequency of cell disorders indicative of malignant disease in oral lichen planus and mild, moderate and severe epithelial dysplasia (A - increased nuclear-cytoplasmic ratio; B - nuclear hyperchromatism; C - irregular chromatin distribution; D - thickened nuclear membrane; E - loss of cell cohesion; F - enlarged nucleoli; G - multinucleate cells; H - cell and nucleus pleomorphism)

The most frequently observed disorders on oral lichen planus cases were increased nuclear-cytoplasmic ratio (92.86%), thickened nuclear membrane (85.71%), and multinucleate cells (85.71%). Increased nuclear-cytoplasmic ratio was also the most frequently encountered disorder in epithelial dysplasia, together with cell and nucleus pleomorphism, seen in 100.00% of the cases regardless of degree of dysplasia (Fig. 1).

The chi-square test showed no statistically significant differences between oral lichen planus and epithelial dysplasia for the following cell disorders: increased nuclear-cytoplasmic ratio, nuclear hyperchromatism, irregular chromatin distribution, and enlarged nucleoli (p>0.05). Nonetheless, this test also revealed that thickened nuclear membrane, loss of cell cohesion, and multinucleate cells are seen more often in lichen planus (p<0.05), while cell and nucleus pleomorphism is more frequently observed in epithelial dysplasia (p<0.05).

## DISCUSSION

Although the World Health Organization (WHO) currently considers oral lichen planus to be a disease that may evolve to cancer^20^, its actual potential to evolve malignantly is the target of much controversy.

Krutchkoff et al.^10^ (1978) reviewed papers published in the literature between 1950 and 1976 on the premalignant nature of oral lichen planus and concluded it was not possible to establish a safe correlation between lichen planus and oral cancer due to the lack of clinical and histopathology data from the analyzed cases. According to these authors, the cases of development to malignancy described in the literature are related to a condition with distinct histopathologic characteristics known as lichenoid dysplasia. This condition manifests characteristics that are similar to the ones encountered in oral lichen planus, but it also presents altered epithelial cell maturation patterns, thus excluding lichen planus from the diagnostic possibilities.

Eisenberg and Krutchkoff^11^ (1992) reported that oral lichen planus is unlikely to evolve malignantly. According to them, many of the cases that eventually evolved to epidermoid carcinoma were actually lichenoid dysplasia cases wrongly diagnosed as lichen planus, given the large number of histopathologic similarities between both conditions, more specifically when in their early stages.

Recent studies are changing the Idea that oral lichen planus and lichenoid dysplasia should be seen as two completely separate entities. Today it is known that alterations to the normal epithelial cell maturation pattern are strongly correlated to lesions evolving malignantly. Therefore, it is lichenoid dysplasia - and not lichen planus - that should be categorized as a condition that may evolve to malignancy. Indeed, the possibility of evolving malignantly reflects a series of cell-intrinsic molecular alterations seen in lichenoid dysplasia ^21^, as reported by Kim et al.^16^ (2001) in their investigation into chromosome 9 monosomy, an important step in the process of malignant development.

Van der Meij et al.^18^ (2003) performed a prospective study in which 62 patients with oral lichen planus and 111 with lichenoid dysplasia were followed up for 6.6 to 72 months. Three (1.7%) of the 173 studied patients evolved to epidermoid carcinoma. All cases that evolved to malignant disease had lichenoid dysplasia.

However, it is worth noticing that even the classic cases of epithelial dysplasia, i.e., the ones without any lichenoid traits, may produce findings similar to oral lichen planus. The results obtained in this study make it evident that the histopathologic diagnosis of oral lichen planus, mainly when differentiated against epithelial dysplasia, is quite difficult, as some cell disorders indicative of malignant disease such as increased nuclear-cytoplasmic ratio, nuclear hyperchromatism, and irregular chromatin distribution may be seen in either of the lesions. On the other hand, they also suggest that cell and nucleus pleomorphism is a highly relevant finding, as it points to lichenoid dysplasia and consequently patients that require closer clinical follow-up.

It should not be neglected that the very chronic inflammatory process present in lichen planus leads to the onset of cell disorders that mimic the ones seen in epithelial dysplasia, without however possessing any malignant connotation.^15^ On the other hand, for Mignogna et al.^22^ (2004), chronic inflammatory processes create a micro-ambience in which cell survival, growth, differentiation and proliferation are impacted, consequently contributing with carcinogenesis, leading such alterations to be thought of as possible indication of malignant development.

The concern around the malignant potential of oral lichen planus is much more related to diagnostic difficulties than to the nature of the condition itself. Notwithstanding the controversy that surrounds this topic, health care workers must be fully aware of the importance of providing close follow-up to patients with lichen planus and other chronic diseases.

## CONCLUSION

Reaching a diagnosis of oral lichen planus is no easy task due to the lack of accurate clinical and histopathologic criteria. Additionally, some cell disorders indicative of malignant disease present in oral lichen planus may also be seen in epithelial dysplasia, thus making diagnosis even harder and consequently emphasizing the relevance of offering long term follow-up to patients with this disease, not because of its malignant potential, but due to possible mistakes made in the initial diagnosis.
